# Contributions of Thyroid Hormone to Cancer Metastasis

**DOI:** 10.3390/biomedicines6030089

**Published:** 2018-08-22

**Authors:** Shaker A. Mousa, Gennadi V. Glinsky, Hung-Yun Lin, Osnat Ashur-Fabian, Aleck Hercbergs, Kelly A. Keating, Paul J. Davis

**Affiliations:** 1 Pharmaceutical Research Institute, Albany College of Pharmacy and Health Sciences, Rensselaer, NY 12144, USA; shaker.mousa@acphs.edu (S.A.M.); linhy@tmu.edu.tw (H.-Y.L.); kelly.keating@acphs.edu (K.A.K.); 2 Institute of Engineering in Medicine, University of California, San Diego, CA 92093, USA; gglinskii@ucsd.edu; 3 PhD Program for Cancer Molecular Biology and Drug Discovery, College of Medical Science and Technology, Taipei Medical University, Taipei 11031, Taiwan; 4 Taipei Cancer Center, Taipei Medical University, Taipei 11031 Taiwan; 5 Traditional Herbal Medicine Research Center of Taipei Medical University Hospital, Taipei 11031, Taiwan; 6 TMU Research Center of Cancer Translational Medicine, Taipei Medical University, Taipei 11031, Taiwan; 7 Department of Human Molecular Genetics and Biochemistry, Tel Aviv University, Tel Aviv 69978, Israel; osnataf@gmail.com; 8 Department of Radiation Oncology, Cleveland Clinic, Cleveland, OH 44195, USA; hercbergs@gmail.com; 9 Department of Medicine, Albany Medical College, Albany, NY 12208, USA

**Keywords:** angiogenesis, cancer, cancer cell genes, epithelial-to-mesenchymal transition (EMT), integrin αvβ3, l-thyroxine, matrix metalloproteinases, metastasis, T4, tetrac, thyroid hormone

## Abstract

Acting at a cell surface receptor on the extracellular domain of integrin αvβ3, thyroid hormone analogues regulate downstream the expression of a large panel of genes relevant to cancer cell proliferation, to cancer cell survival pathways, and to tumor-linked angiogenesis. Because αvβ3 is involved in the cancer cell metastatic process, we examine here the possibility that thyroid hormone as l-thyroxine (T4) and the thyroid hormone antagonist, tetraiodothyroacetic acid (tetrac), may respectively promote and inhibit metastasis. Actions of T4 and tetrac that are relevant to cancer metastasis include the multitude of synergistic effects on molecular levels such as expression of matrix metalloproteinase genes, angiogenesis support genes, receptor tyrosine kinase (*EGFR*/*ERBB2*) genes, specific microRNAs, the epithelial–mesenchymal transition (EMT) process; and on the cellular level are exemplified by effects on macrophages. We conclude that the thyroid hormone-αvβ3 interaction is mechanistically linked to cancer metastasis and that modified tetrac molecules have antimetastatic activity with feasible therapeutic potential.

## 1. Introduction

Evidence that is preclinical [1,2,3,4,5,6] and clinical [7,8,9,10] indicates that thyroid hormone can support tumor cell proliferation, antiapoptosis, and cancer-associated angiogenesis. Certain of these actions of the hormone are initiated at a thyroid hormone–tetraiodothyroacetic acid (tetrac) receptor on the extracellular domain of integrin αvβ3 [11,12,13]. The integrin is expressed primarily by cancer cells and by dividing endothelial cells that are related to cancer [12]. l-Thyroxine (T4) is the primary ligand of this receptor and is effective at physiological concentrations. In contrast, 3,5,3′-triiodo-l-thyronine (T3) is the primary intracellular thyroid hormone analogue and is the principal ligand of the nuclear receptors for thyroid hormone (TRs) [11,14,15]. Actions of thyroid hormone that are initiated by the binding of T3 at TRs are designated genomic, whereas effects of thyroid hormones—chiefly, T4—that are initiated outside the nucleus, e.g., at the plasma membrane or mitochondria [11], are described as nongenomic. However, substantial evidence exists that T4 can act at the plasma membrane to promote via downstream signal transduction a substantial panel of intranuclear events, including specific gene transcription and activation (site-specific phosphorylation) of TRs [11,12,13].

**Figure 1:**
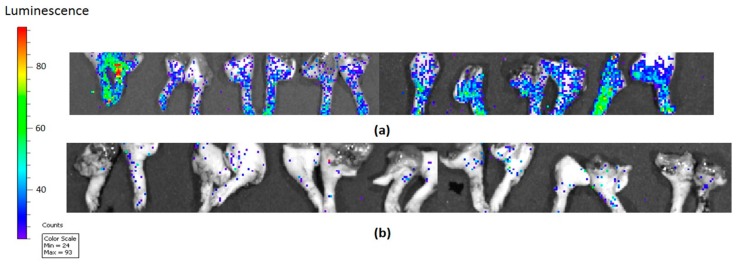
Tetrac covalently linked to polyethylene glycol (PEG) (named P-bi-TAT) was used as treatment in a breast cancer mouse model compared to control and shows the elimination of limb bone metastasis, implicating αvβ3 in the thyroid hormone-metastasis pathway [19]. Images shown were obtained with an In Vitro Imaging System (IVIS); vertical luminescence color bar estimates viability, ranging from nonviable (blue) to fully viable (red). (**a**) Metastatic luminescent signals of (MCF7-luc) breast cancer control (phosphate buffered saline) in mouse limbs (bones) in an orthotopic mouse model (female nude mice); (**b**) Metastatic luminescent signals of (MCF7-luc) breast cancer after P-bi-TAT subcutaneous treatment with 3 mg/kg TAT equivalent daily for 18 days shows the elimination of metastasis.

Integrin αvβ3 and its T4 receptor have also been implicated in the process of cancer metastasis [16,17,18]. Tetrac in its small molecule formulation acts as a T4 antagonist at the αvβ3 integrin [12]. Tetrac covalently linked to polyethylene glycol (PEG) to prolong its actions at αvβ3 receptor has eliminated breast cancer xenografts that have metastasized to bone (Figure 1). Such direct evidence of the potent antimetastatic activity has prompted this brief review of actions of thyroid hormone and/or tetrac that may be relevant to the process of cancer cell metastasis. We conclude that thyroid hormone as T4 appears to have access via αvβ3 signaling to a substantial number of molecular mechanisms that contribute to metastatic dissemination of cancer cells.

## 2. Cancer Metastasis-Relevant Molecular Mechanisms of Thyroid Hormone Action

### 2.1. Matrix Metalloproteinase (MMP) Gene Expression and Metastasis

MMP-2 and MMP-9 have been implicated in metastasis [20,21,22]. The processes of liberating cancer cells from a primary tumor or permitting the seating of circulating tumor cells at a distant site depend upon the solubilizing of the extracellular matrix at the primary lesion and metastatic sites, respectively. MMPs are essential to the process of dissolving of the extracellular matrix during cancer metastasis. A nanopharmaceutical formulation of tetrac as chemically modified nanoparticles (Nano-diamino-tetrac, NDAT) was engineered to extend its life in the pericellular microenvironment of cancer cells or blood vessel cells, and it has been shown that NDAT administration potently downregulates expression of *MMP-2* and *MMP-9* genes [13,23]. These observations relate directly to the anticancer activity of NDAT, and thyroid hormone has also been shown to enhance expression and activity of MMPs, e.g., *MMP-9* [21].

### 2.2. Angiogenesis and Metastasis

The rapid growth of primary tumors and of metastases depends upon an ample local circulation, and much attention in cancer treatment has been directed at vascular targets, such as vascular growth factors (vascular endothelial growth factor, VEGF; basic fibroblast growth factor (bFGF)) or their receptors on endothelial cells. Tumor-supporting blood vessels may be porous, reflecting an angiogenic process that is so rapid that vessels are not fully completed.

The proangiogenic properties of physiologic levels of T4 have been demonstrated in models such as the fertilized chick egg chorioallantoic membrane (CAM) model [24,25]. Notably, T3 also is proangiogenic, but at higher-than-physiologic concentrations. Thyroid hormone affects transcription of the *VEGF* and *bFGF* genes, and of the platelet-derived growth factor (*PDGF*) gene [2,26]. Epidermal growth factor (EGF) also has proangiogenic activity, and expression of the *EGFR* gene is stimulated by thyroid hormone [13]. It may also be noted that modified tetrac (NDAT) affects the activity in the CAM assay of small angiogenic factors, such as angiotensin-2 (Ang-2), lipopolysaccharide (LPS), and bradykinin [27]. Again, the implication here is not only that NDAT has significant antiangiogenic potency, but that T4 manifests proangiogenic activity and may also act at the αvβ3 to support angiogenesis associated with primary tumors and metastatic-relevant angiogenesis.

### 2.3. microRNAs (miRs) and Metastasis

A substantial literature has documented the critical contributions of specific miRs to the metastatic process [28]. The actions of thyroid hormone analogues on expression of specific miRs have not been extensively studied as they may relate to metastasis, but miR-21 and miR-15A are examples of miRs relevant to metastasis and whose expression is differentially regulated by iodothyronines [13].

miR-15A is antiangiogenic and decreases osteosarcoma cell invasiveness [29] and is upregulated by tetrac at αvβ3. The same miR also acts to reduce metastasis by inhibiting multiple components of the TGFβ pathway [30], which is discussed in more detail in a later section.

The roles of miR-21 in cancer cell biology are numerous [31]. miR-21 increases the metastatic potential of cancer cells, e.g., non-small cell lung carcinoma [32], and this in part is due to its proangiogenic properties. The abundance of this miR in tumor cells may be decreased by up to 50% in certain tumor cells by tetrac products [13]. Thus, thyroid hormone analogues have actions relevant to metastasis that are initiated at αvβ3 that are mediated by miRs. The effects of tetrac on miRs appear highly consistent with its antitumor and antimetastatic activity. Whether T4 acts at the integrin to regulate these actions and support metastasis is not yet known.

### 2.4. Epithelial–Mesenchymal Transition (EMT) and Mechanism of Metastasis

The link of thyroid hormone, particularly T4, to the EMT has recently been examined [17]. EMT is a cog in the mechanism of metastasis, accounting for the genesis of cells capable of metastasizing [33]. Acing via the hormone receptor on integrin αvβ3, thyroid hormone has been shown to promote EMT. The basis of the process includes induction of β-catenin by the hormone, as well as certain downstream molecular targets of the catenin. It will be of interest to investigate the effects of tetrac and its nanopharmaceutical formulations on EMT, but it is reasonable to expect their potent inhibitory actions on the EMT process.

### 2.5. Transforming Growth Factor β (TGFβ) and Metastasis

The modulation of expression of this gene in normal tissues has for many years been understood to be a function of steroid and thyroid hormone actions [34]. In such tissues, cell proliferation, differentiation, and functional behavior are subject to regulation by TGFβ. T4 has been shown to potentiate TGFβ-induced normal airway smooth muscle cell proliferation, and this potentiation is mediated by the hormone receptor on integrin αvβ3 [35]. The synergism of T4 and TGFβ is blocked by tetrac. In certain noncancer cells, T3 has been shown to inhibit TGFβ action [36]. This hormonal action appears to be nuclear thyroid hormone receptor (TR)-dependent and requires nanomolar concentrations of T3 that are above its physiologic level.

The contributions of dysregulated TGFβ pathway to the processes of oncogenic transformation and of metastasis are also now widely appreciated and have recently been reviewed [37]. In the setting of cancer, the growth factor may contribute to metastasis via actions on angiogenesis, lymphangiogenesis, and transendothelial migration of prometastatic cancer cells [37]. From the observations of Dekkers et al. cited above [35], we know that T4 can support cell proliferation via αvβ3 and we propose that T4 may also do so in the setting of cancer and perhaps act as a potent prometastatic agent. This possibility requires investigation, but from the observations presented above in Figure 1, it is clear that a tetrac analogue acting at the integrin receptor—an inhibitor of actions of T4 at αvβ3—antagonizes metastasis.

### 2.6. EGF Receptor (EGFR), Metastasis and Angiogenesis

Expression of the EGFR gene by tumor cells is associated with drug resistance, metastasis [38], and angiogenic support of metastases. Induction of transcription of *EGFR* by T4—and downregulation of *EGFR* expression by NDAT—is included below in the ‘Selected Cancer Driver Genes’ section. Because of its association with drug resistance and metastasis, EGFR protein is an established chemotherapeutic target. The effects on *EGFR* expression of both T4 and tetrac are highly consistent with their proposed roles as promoter and inhibitor of cancer metastasis, respectively.

### 2.7. Tumor-Associated Macrophages (TAMs) and Metastasis

The TAM is a part of the profile of the premetastatic component of malignant tumors [33]. In inflamed tumors, it may not be feasible to distinguish the inflammation-related macrophage from the macrophage destined to participate in the metastatic process. What we note here is that the macrophages involved in phagocytosis of microorganisms are subject to regulation by thyroid hormone via integrin αvβ3 [39,40]. Phagocytosis is recognized to be a shared property of macrophages and metastatic cancer cells [33] and appears to be supported by thyroid hormone.

## 3. Selected Cancer Driver Genes, Thyroid Hormone Analogues and Metastases

Myeloid cell leukemia-1 (*MCL1*) gene expression is associated with metastases of various cancers [41,42], and tetrac-containing NDAT downregulates this gene [43]. DNA fragmentation factor subunit α is encoded by the *DFFA* gene; this factor supports apoptosis and thus is seen to be antimetastatic [44]. Expression of this gene is increased by NDAT [13]. The *EGFR* gene was mentioned above as a target of NDAT. Mutation of the gene is linked to distant metastases, e.g., of lung cancer [45], and transcription of this gene is significantly reduced by NDAT [13].

Microarray experiments of cancer driver gene expression in human glioblastoma cells exposed to a new tetrac-containing agent, P-bi-TAT, revealed that expression of at least nine cancer driver genes is significantly downregulated in primary human glioblastoma multiforme cells treated with P-bi-TAT, including *AKT1*, *AKT2*, *CD4*, *ERBB2*, *HRAS*, *IDH2*, *KIT*, *MAP2K7* (*MKK7*; *JNKK2*), and *MLST8* (*GBL*; *LST8*). Of note, *ERBB2* transcription was downregulated 2-fold by the agent [46]. Overexpression of *ERBB2* has been associated clinically with shorter time to first metastasis in breast cancer [47] and thus chemotherapeutic suppression of transcription of this gene would appear to be desirable. However, progression-free survival can paradoxically be lengthened in the setting of overexpression of *ERBB2* [47], suggesting more complex mechanisms of pathogenesis. In the context of the present contribution, it is important to highlight the convincing clinical and experimental evidence linking at least seven of these P-bi-TAT-suppressed cancer driver genes with metastatic disease in patients diagnosed with multiple types of malignancies, as detailed in Table 1.

Expression of cyclin-dependent kinase 4/6 (*CDK4/6*) may be increased in metastatic cancer, for example, breast carcinoma metastatic to the brain [79], and thus the gene is a therapeutic target. P-bi-TAT reduces by 2-fold the transcription of this gene [46]. The actions of P-bi-TAT on *ERBB2* and *CDK4/6* have several implications. First, because the action of the agent is exclusively at integrin αvβ3, the thyroid hormone-tetrac receptor is involved in control of the expression of these genes. Second, the action of P-bi-TAT is consistent with the possibility that T4 upregulates transcription of the gene, but this issue has not yet been examined directly.

The *KIT* or *CD117* gene codes for a receptor tyrosine kinase that is frequently overexpressed or mutated in breast cancer metastatic to brain [80]. *KIT* is also a member of the cancer driver gene panel whose transcription is downregulated by P-bi-TAT [46]. Thus, the cell surface receptor for T4 and tetrac is a factor regulating the contribution of the mutated or overexpressed gene to cancer progression and metastasis.

## 4. Fibronectin and Metastasis

Thyroid hormone is known to control fibronectin expression via a signal transduction pathway involving phosphatidylinositol 3-kinase (PI3K)/Akt and hypoxia-inducible factor-1α (HIF-1α) [81]. This pathway is regulated from the thyroid hormone receptor on integrin αvβ3 [15]. Plasma fibronectin has been shown to support metastasis, e.g., of lung cancer cells, via induction of tumor cell invasiveness that depends upon activation of integrin αvβ3 [82]. Thus, effects of thyroid hormone on fibronectin expression seems consistent with the proposed role in promoting cancer metastasis.

## 5. Discussion

The process of metastasis at the molecular level is now subject to a substantial number of new insights. It is clear that a myriad of factors regulated by tumors and by the tumor host contribute to the metastatic process [33,83,84,85]. As we have noted, actions of thyroid hormone analogues initiated at a receptor site on integrin αvβ3 differentially affect a wide variety of tumor-relevant processes, including cell proliferation, regulation of apoptosis, and control of angiogenesis that is linked to the tumor development and metastatic progression [11,12,13].

The positive impact of spontaneous or medically induced hypothyroidism on cancer patient survival [7,8,9,10,86], particularly with the specific withdrawal of T4 in the setting of euthyroid hypothyroxinemia [87], complements extensive in vitro studies of the proliferative action of thyroid hormone on tumor cells. If hypothyroidism/hypothyroxinemia result in prolongation of survival in cancer patients, then we have to suspect that metastases, as well as primary tumors, are being affected by the altered thyroid state. This caused us in the present brief review to search for evidence in the literature of links between thyroid hormone and the abundance or activity of host and tumor factors that have been shown to support the metastatic process.

There is an abundance of such evidence and much of it is consistent with the involvement of the thyroid hormone receptor on the extracellular domain of integrin αvβ3 on metastatic cancer cells or on endothelial cells. The primary thyroid hormone ligand of this receptor is T4 and unmodified or modified tetrac also acts here. T4 appears to support metastasis and, as reviewed here, tetrac blocks the actions of T4 that are metastasis-promoting. The actions of thyroid hormone analogues on expression of genes that have been associated with metastasis are differential—that is, T4 acts to up- or downregulate gene expression in order to support metastasis. Consistent with this concept, the effects of tetrac and/or its nanopharmaceutical formulations are also differential, but the mirror-image of T4 actions. That is, tetrac inhibits the expression of genes promoting metastasis and T4 activates the expression of genes inhibiting metastasis.

In this review we have emphasized the contributions of thyroid hormone as T4 to the process of metastasis. Others have described the inhibitory effects of thyroid hormone as T3 on cancer growth [88,89,90,91]. The concentrations of T3 used in the studies cited are supraphysiologic (e.g., 10^−8^ M), but we have shown that physiologic concentrations of T3 in clinical cancer settings can be associated with tumor growth arrest and sometimes with tumor shrinkage [87]. Thus, the roles of T4 and T3 are very different in cancer.

We have pointed out that there are inconsistencies in the actions of thyroid hormone on expression of certain genes that relate to metastasis; an example of this is thyroid hormone effects on TGFβ expression on several cell lines. Even here, however, there may be a pattern of coherence, in that the hormone suppresses TGFβ expression in noncancer cells and enhances it in the limited number of tumor cells in which this activity has been examined so far.

## 6. Conclusions

Multiple lines of well-documented experimental evidence support the concept that thyroid hormone as T4 appears to have access via αvβ3 signaling to a substantial number of molecular mechanisms that promote cancer metastasis. Consequently, T4 antagonist tetrac, and particularly its nanopharmaceutical formulations, have a significant therapeutic potential as potent antimetastatic agents.

## Figures and Tables

**Table 1:** Examples of cancer driver genes implicated in development of metastatic disease in multiple types of human malignancies, expression of which was significantly repressed by P-bi-TAT. References cited report clinical and experimental evidence linking these genes with development of metastasis in patients diagnosed with different cancer types.

Gene	Metastasis of Cancer Type	References
*AKT1*	breast cancer; colorectal cancer; osteosarcoma; esophageal squamous cell carcinoma; tongue squamous cell carcinoma	[48,49,50,51,52,53,54,55]
*AKT2*	breast cancer; colorectal cancer; multiple cancer types	[51,56,57,58]
*ERBB2*	prostate cancer; breast cancer; gastric cancer	[59,60,61,62]
*HRAS*	breast cancer	[48]
*IDH2*	intrahepatic cholangiocarcinoma (biliary cancer); osteosarcoma (loss of tumor suppressor function); hepatocellular carcinoma; low grade diffuse glioma	[63,64,65,66]
*KIT*	breast cancer; prostate cancer; colorectal cancer; melanoma; gastrointestinal stromal tumors	[67,68,69,70,71,72,73,74,75]
*MAP2K7* (*MKK7*)	lung cancer; colon cancer; pancreatic cancer	[76,77,78]

## References

[1] Pinto M., Soares P., Ribatti D. (2011). Thyroid hormone as a regulator of tumor induced angiogenesis. Cancer Lett..

[2] Davis P.J., Sudha T., Lin H.Y., Mousa S.A. (2015). Thyroid hormone, hormone analogs, and angiogenesis. Compr. Physiol..

[3] Cremaschi G.A., Cayrol F., Sterle H.A., Diaz Flaque M.C., Barreiro Arcos M.L. (2016). Thyroid hormones and their membrane receptors as therapeutic targets for T cell lymphomas. Pharmacol. Res..

[4] Lin H.Y., Chin Y.T., Yang Y.C., Lai H.Y., Wang-Peng J., Liu L.F., Tang H.Y., Davis P.J. (2016). Thyroid hormone, cancer, and apoptosis. Compr. Physiol..

[5] Shinderman-Maman E., Cohen K., Weingarten C., Nabriski D., Twito O., Baraf L., Hercbergs A., Davis P.J., Werner H., Ellis M. (2016). The thyroid hormone-αvβ3 integrin axis in ovarian cancer: Regulation of gene transcription and MAPK-dependent proliferation. Oncogene.

[6] Chin Y.T., Wei P.L., Ho Y., Nana A.W., Changou C.A., Chen Y.R., Yang Y.S., Hsieh M.T., Hercbergs A., Davis P.J. (2018). Thyroxine inhibits resveratrol-caused apoptosis by PD-L1 in ovarian cancer cells. Endocr. Relat. Cancer.

[7] Schmidinger M., Vogl U.M., Bojic M., Lamm W., Heinzl H., Haitel A., Clodi M., Kramer G., Zielinski C.C. (2011). Hypothyroidism in patients with renal cell carcinoma: Blessing or curse?. Cancer.

[8] Bailey E.B., Tantravahi S.K., Poole A., Agarwal A.M., Straubhar A.M., Batten J.A., Patel S.B., Wells C.E., Stenehjem D.D., Agarwal N. (2015). Correlation of degree of hypothyroidism with survival outcomes in patients with metastatic renal cell carcinoma receiving vascular endothelial growth factor receptor tyrosine kinase inhibitors. Clin. Genitourin. Cancer.

[9] Cristofanilli M., Yamamura Y., Kau S.W., Bevers T., Strom S., Patangan M., Hsu L., Krishnamurthy S., Theriault R.L., Hortobagyi G.N. (2005). Thyroid hormone and breast carcinoma. Primary hypothyroidism is associated with a reduced incidence of primary breast carcinoma. Cancer.

[10] Nelson M., Hercbergs A., Rybicki L., Strome M. (2006). Association between development of hypothyroidism and improved survival in patients with head and neck cancer. Arch. Otolaryngol. Head Neck Surg..

[11] Cheng S.Y., Leonard J.L., Davis P.J. (2010). Molecular aspects of thyroid hormone actions. Endocr. Rev..

[12] Davis P.J., Goglia F., Leonard J.L. (2016). Nongenomic actions of thyroid hormone. Nat. Rev. Endocrinol..

[13] Davis P.J., Glinsky G.V., Lin H.-Y., Leith J.T., Hercbergs A., Tang H.-Y., Ashur-Fabian O., Incerpi S., Mousa S.A. (2014). Cancer cell gene expression modulated from plasma membrane integrin αvβ3 by thyroid hormone and nanoparticulate tetrac. Front. Endocrinol..

[14] Lin H.Y., Landersdorfer C.B., London D., Meng R., Lim C.U., Lin C., Lin S., Tang H.Y., Brown D., Van Scoy B. (2011). Pharmacodynamic modeling of anti-cancer activity of tetraiodothyroacetic acid in a perfused cell culture system. PLoS Comput. Biol..

[15] Lin H.Y., Sun M., Tang H.Y., Lin C., Luidens M.K., Mousa S.A., Incerpi S., Drusano G.L., Davis F.B., Davis P.J. (2009). l-thyroxine vs. 3,5,3’-triiodo-l-thyronine and cell proliferation: Activation of mitogen-activated protein kinase and phosphatidylinositol 3-kinase. Am. J. Physiol. Cell Physiol..

[16] Shi S., Zhou M., Li X., Hu M., Li C., Li M., Sheng F., Li Z., Wu G., Luo M. (2016). Synergistic active targeting of dually integrin αvβ3/CD44-targeted nanoparticles to B16F10 tumors located at different sites of mouse bodies. J. Control. Release.

[17] Weingarten C., Jenudi Y., Tshuva R.Y., Moskovich D., Alfandari A., Hercbergs A., Davis P.J., Ellis M., Ashur-Fabian O. (2018). The interplay between epithelial-mesenchymal transition (EMT) and the thyroid hormones-αvβ3 axis in ovarian cancer. Horm. Cancer.

[18] Zhang P., Chen L., Song Y., Li X., Sun Y., Xiao Y., Xing Y. (2016). Tetraiodothyroacetic acid and transthyretin silencing inhibit pro-metastatic effect of l-thyroxin in anoikis-resistant prostate cancer cells through regulation of MAPK/ERK pathway. Exp. Cell Res..

[19] Mousa S.A., Davis P.J. Pro- and anti-metastatic properties of specific thyroid hormone analogues.

[20] Tauro M., Lynch C.C. (2018). Cutting to the chase: How matrix metalloproteinase-2 activity controls breast-cancer-to-bone metastasis. Cancers.

[21] Hong M., Cheng H., Song L., Wang W., Wang Q., Xu D., Xing W. (2018). Wogonin suppresses the activity of matrix metalloproteinase-9 and inhibits migration and invasion in human hepatocellular carcinoma. Molecules.

[22] Cai X., Zhu H., Li Y. (2017). Pkcz, MMP-2 and MMP-9 expression in lung adenocarcinoma and association with a metastatic phenotype. Mol. Med. Rep..

[23] Cohen K., Flint N., Shalev S., Erez D., Baharal T., Davis P.J., Hercbergs A., Ellis M., Ashur-Fabian O. (2014). Thyroid hormone regulates adhesion, migration and matrix metalloproteinase 9 activity via αvβ3 integrin in myeloma cells. Oncotarget.

[24] Davis F.B., Mousa S.A., O’Connor L., Mohamed S., Lin H.Y., Cao H.J., Davis P.J. (2004). Proangiogenic action of thyroid hormone is fibroblast growth factor-dependent and is initiated at the cell surface. Circ. Res..

[25] Mousa S.S., Mousa S.S., Mousa S.A. (2005). Effect of resveratrol on angiogenesis and platelet/fibrin-accelerated tumor growth in the chick chorioallantoic membrane model. Nutr. Cancer.

[26] Chen J., Ortmeier S.B., Savinova O.V., Nareddy V.B., Beyer A.J., Wang D., Gerdes A.M. (2012). Thyroid hormone induces sprouting angiogenesis in adult heart of hypothyroid mice through the PDGF-Akt pathway. J. Cell. Mol. Med..

[27] Mousa S.A., Lin H.Y., Tang H.Y., Hercbergs A., Luidens M.K., Davis P.J. (2014). Modulation of angiogenesis by thyroid hormone and hormone analogues: Implications for cancer management. Angiogenesis.

[28] Lima C.R., Gomes C.C., Santos M.F. (2017). Role of microRNAs in endocrine cancer metastasis. Mol. Cell. Endocrinol..

[29] Leng J., Song Q., Zhao Y., Wang Z. (2018). Mi-R15a represses cancer cell migration and invasion under conditions of hypoxia by targeting and downregulating Bcl-2 expression in human osteosarcoma cells. Int. J. Oncol..

[30] Jin W., Chen F., Wang K., Song Y., Fei X., Wu B. (2018). miR-15a/miR-16 cluster inhibits invasion of prostate cancer cells by suppressing TGF-β signaling pathway. Biomed. Pharmacother..

[31] Pfeffer S.R., Yang C.H., Pfeffer L.M. (2015). The role of miR-21 in cancer. Drug Dev. Res..

[32] Liu Z.L., Wang H., Liu J., Wang Z.X. (2013). MicroRNA-21 (miR-21) expression promotes growth, metastasis, and chemo- or radioresistance in non-small cell lung cancer cells by targeting PTEN. Mol. Cell. Biochem..

[33] Seyfried T.N., Huysentruyt L.C. (2013). On the origin of cancer metastasis. Crit. Rev. Oncog..

[34] Koli K., Keski-Oja J. (1996). Transforming growth factor-β system and its regulation by members of the steroid-thyroid hormone superfamily. Adv. Cancer Res..

[35] Dekkers B.G., Naeimi S., Bos I.S., Menzen M.H., Halayko A.J., Hashjin G.S., Meurs H. (2015). l-thyroxine promotes a proliferative airway smooth muscle phenotype in the presence of TGF-β1. Am. J. Physiol. Lung Cell. Mol. Physiol..

[36] Alonso-Merino E., Martin Orozco R., Ruiz-Llorente L., Martinez-Iglesias O.A., Velasco-Martin J.P., Montero-Pedrazuela A., Fanjul-Rodriguez L., Contreras-Jurado C., Regadera J., Aranda A. (2016). Thyroid hormones inhibit TGF-β signaling and attenuate fibrotic responses. Proc. Natl. Acad. Sci. USA.

[37] Bellomo C., Caja L., Moustakas A. (2016). Transforming growth factor β as regulator of cancer stemness and metastasis. Br. J. Cancer.

[38] Wang Z., Candelora C. (2017). In vitro enzyme kinetics analysis of EGFR. Methods Mol. Biol..

[39] Chen Y., Sjolinder M., Wang X., Altenbacher G., Hagner M., Berglund P., Gao Y., Lu T., Jonsson A.B., Sjolinder H. (2012). Thyroid hormone enhances nitric oxide-mediated bacterial clearance and promotes survival after meningococcal infection. PLoS ONE.

[40] De Vito P., Balducci V., Leone S., Percario Z., Mangino G., Davis P.J., Davis F.B., Affabris E., Luly P., Pedersen J.Z. (2012). Nongenomic effects of thyroid hormones on the immune system cells: New targets, old players. Steroids.

[41] Duquette M., Sadow P.M., Husain A., Sims J.N., Antonello Z.A., Fischer A.H., Song C., Castellanos-Rizaldos E., Makrigiorgos G.M., Kurebayashi J. (2015). Metastasis-associated MCL1 and P16 copy number alterations dictate resistance to vemurafenib in a BRAFV600E patient-derived papillary thyroid carcinoma preclinical model. Oncotarget.

[42] Lee W.S., Park Y.L., Kim N., Oh H.H., Son D.J., Kim M.Y., Oak C.Y., Chung C.Y., Park H.C., Kim J.S. (2015). Myeloid cell leukemia-1 regulates the cell growth and predicts prognosis in gastric cancer. Int. J. Oncol..

[43] Glinskii A.B., Glinsky G.V., Lin H.Y., Tang H.Y., Sun M., Davis F.B., Luidens M.K., Mousa S.A., Hercbergs A.H., Davis P.J. (2009). Modification of survival pathway gene expression in human breast cancer cells by tetraiodothyroacetic acid (tetrac). Cell Cycle.

[44] Fawzy M.S., Toraih E.A., Ibrahiem A., Abdeldayem H., Mohamed A.O., Abdel-Daim M.M. (2017). Evaluation of miRNA-196a2 and apoptosis-related target genes: ANXA1, DFFA and PDCD4 expression in gastrointestinal cancer patients: A pilot study. PLoS ONE.

[45] Fujimoto D., Ueda H., Shimizu R., Kato R., Otoshi T., Kawamura T., Tamai K., Shibata Y., Matsumoto T., Nagata K. (2014). Features and prognostic impact of distant metastasis in patients with stage IV lung adenocarcinoma harboring EGFR mutations: Importance of bone metastasis. Clin. Exp. Metastasis.

[46] Glinksy G.V., Davis P.J. Differential cancer driver gene expression by P-bi-TAT, a PEGylated modification of the thyroid hormone analogue, tetraiodothyroacetic acid (tetrac).

[47] Fuchs E.M., Kostler W.J., Horvat R., Hudelist G., Kubista E., Attems J., Zielinski C.C., Singer C.F. (2014). High-level ERBB2 gene amplification is associated with a particularly short time-to-metastasis, but results in a high rate of complete response once trastuzumab-based therapy is offered in the metastatic setting. Int. J. Cancer.

[48] Donovan C.A., Pommier R.F., Schillace R., O’Neill S., Muller P., Alabran J.L., Hansen J.E., Murphy J.A., Naik A.M., Vetto J.T. (2013). Correlation of breast cancer axillary lymph node metastases with stem cell mutations. JAMA Surg..

[49] Wei W.T., Nian X.X., Wang S.Y., Jiao H.L., Wang Y.X., Xiao Z.Y., Yang R.W., Ding Y.Q., Ye Y.P., Liao W.T. (2017). MiR-422a inhibits cell proliferation in colorectal cancer by targeting AKT1 and MAPK1. Cancer Cell Int..

[50] Qi C., Chen Y., Zhou Y., Huang X., Li G., Zeng J., Ruan Z., Xie X., Zhang J. (2018). Delineating the underlying molecular mechanisms and key genes involved in metastasis of colorectal cancer via bioinformatics analysis. Oncol. Rep..

[51] Sahlberg S.H., Mortensen A.C., Haglof J., Engskog M.K.R., Arvidsson T., Pettersson C., Glimelius B., Stenerlow B., Nestor M. (2017). Different functions of AKT1 and AKT2 in molecular pathways, cell migration and metabolism in colon cancer cells. Int. J. Oncol..

[52] Zou Q., Xiao X., Liang Y., Peng L., Guo Z., Li W., Yu W. (2018). MiR-19a-mediated downregulation of RhoB inhibits the dephosphorylation of AKT1 and induces osteosarcoma cell metastasis. Cancer Lett..

[53] Li B., Xu W.W., Lam A.K.Y., Wang Y., Hu H.F., Guan X.Y., Qin Y.R., Saremi N., Tsao S.W., He Q.Y. (2017). Significance of PI3K/AKT signaling pathway in metastasis of esophageal squamous cell carcinoma and its potential as a target for anti-metastasis therapy. Oncotarget.

[54] Mao Y., Li L., Liu J., Wang L., Zhou Y. (2016). Mir-495 inhibits esophageal squamous cell carcinoma progression by targeting Akt1. Oncotarget.

[55] Ji M.Y., Wang W., Yan W.X., Chen D., Ding X.Q., Wang A.X. (2017). Dysregulation of AKT1, a miR-138 target gene, is involved in the migration and invasion of tongue squamous cell carcinoma. J. Oral. Pathol. Med..

[56] Riggio M., Perrone M.C., Polo M.L., Rodriguez M.J., May M., Abba M., Lanari C., Novaro V. (2017). AKT1 and AKT2 isoforms play distinct roles during breast cancer progression through the regulation of specific downstream proteins. Sci. Rep..

[57] Agarwal E., Robb C.M., Smith L.M., Brattain M.G., Wang J., Black J.D., Chowdhury S. (2017). Role of Akt2 in regulation of metastasis suppressor 1 expression and colorectal cancer metastasis. Oncogene.

[58] Honardoost M., Rad S. (2018). Triangle of AKT2, miRNA, and tumorigenesis in different cancers. Appl. Biochem. Biotechnol..

[59] Tome-Garcia J., Li D., Ghazaryan S., Shu L., Wu L. (2014). ERBB2 increases metastatic potentials specifically in androgen-insensitive prostate cancer cells. PLoS ONE.

[60] Ferracin M., Bassi C., Pedriali M., Pagotto S., D’Abundo L., Zagatti B., Corra F., Musa G., Callegari E., Lupini L. (2013). MiR-125b targets erythropoietin and its receptor and their expression correlates with metastatic potential and ERBB2/HER2 expression. Mol. Cancer.

[61] Worzfeld T., Swiercz J.M., Looso M., Straub B.K., Sivaraj K.K., Offermanns S. (2012). ErbB-2 signals through Plexin-B1 to promote breast cancer metastasis. J. Clin. Investig..

[62] Bayrak M., Olmez O.F., Kurt E., Cubukcu E., Evrensel T., Kanat O., Manavoglu O. (2013). Prognostic significance of c-erbB2 overexpression in patients with metastatic gastric cancer. Clin. Transl. Oncol..

[63] Saha S.K., Parachoniak C.A., Ghanta K.S., Fitamant J., Ross K.N., Najem M.S., Gurumurthy S., Akbay E.A., Sia D., Cornella H. (2014). Mutant IDH inhibits HNF-4α to block hepatocyte differentiation and promote biliary cancer. Nature.

[64] Yi W.R., Li Z.H., Qi B.W., Ernest M.E., Hu X., Yu A.X. (2016). Downregulation of IDH2 exacerbates the malignant progression of osteosarcoma cells via increased NF-κB and MMP-9 activation. Oncol. Rep..

[65] Tian G.Y., Zang S.F., Wang L., Luo Y., Shi J.P., Lou G.Q. (2015). Isocitrate dehydrogenase 2 suppresses the invasion of hepatocellular carcinoma cells via matrix metalloproteinase 9. Cell. Physiol. Biochem..

[66] Fu Y., Zheng S., Zheng Y., Huang R., An N., Liang A., Hu C. (2012). Glioma derived isocitrate dehydrogenase-2 mutations induced up-regulation of HIF-1α and β-catenin signaling: Possible impact on glioma cell metastasis and chemo-resistance. Int. J. Biochem. Cell Biol..

[67] Kashiwagi S., Yashiro M., Takashima T., Aomatsu N., Kawajiri H., Ogawa Y., Onoda N., Ishikawa T., Wakasa K., Hirakawa K. (2013). c-Kit expression as a prognostic molecular marker in patients with basal-like breast cancer. Br. J. Surg..

[68] Jansson S., Bendahl P.O., Grabau D.A., Falck A.K., Ferno M., Aaltonen K., Ryden L. (2014). The three receptor tyrosine kinases c-KIT, VEGFR2 and PDGFRα, closely spaced at 4q12, show increased protein expression in triple-negative breast cancer. PLoS ONE.

[69] Kuonen F., Laurent J., Secondini C., Lorusso G., Stehle J.C., Rausch T., Faes-Van’t Hull E., Bieler G., Alghisi G.C., Schwendener R. (2012). Inhibition of the Kit ligand/c-Kit axis attenuates metastasis in a mouse model mimicking local breast cancer relapse after radiotherapy. Clin. Cancer Res..

[70] Mainetti L.E., Zhe X., Diedrich J., Saliganan A.D., Cho W.J., Cher M.L., Heath E., Fridman R., Kim H.R., Bonfil R.D. (2015). Bone-induced c-kit expression in prostate cancer: A driver of intraosseous tumor growth. Int. J. Cancer.

[71] Spahn M., Kneitz S., Scholz C.J., Stenger N., Rudiger T., Strobel P., Riedmiller H., Kneitz B. (2010). Expression of microRNA-221 is progressively reduced in aggressive prostate cancer and metastasis and predicts clinical recurrence. Int. J. Cancer.

[72] Wiesner C., Nabha S.M., Dos Santos E.B., Yamamoto H., Meng H., Melchior S.W., Bittinger F., Thuroff J.W., Vessella R.L., Cher M. (2008). C-kit and its ligand stem cell factor: Potential contribution to prostate cancer bone metastasis. Neoplasia.

[73] Tan J., Yang S., Shen P., Sun H., Xiao J., Wang Y., Wu B., Ji F., Yan J., Xue H. (2015). C-kit signaling promotes proliferation and invasion of colorectal mucinous adenocarcinoma in a murine model. Oncotarget.

[74] Zhan Y., Guo J., Yang W., Goncalves C., Rzymski T., Dreas A., Zylkiewicz E., Mikulski M., Brzozka K., Golas A. (2017). MNK1/2 inhibition limits oncogenicity and metastasis of KIT-mutant melanoma. J. Clin. Investig..

[75] Sanchez-Hidalgo J.M., Duran-Martinez M., Molero-Payan R., Rufian-Pena S., Arjona-Sanchez A., Casado-Adam A., Cosano-Alvarez A., Briceno-Delgado J. (2018). Gastrointestinal stromal tumors: A multidisciplinary challenge. World J. Gastroenterol..

[76] Qiu F.M., Yang L., Lu X.X., Chen J.S., Wu D., Wei Y.F., Nong Q.Q., Zhang L.S., Fang W.X., Chen X.L. (2016). The MKK7 p.Glu116Lys rare variant serves as a predictor for lung cancer risk and prognosis in Chinese. PLoS Genet..

[77] Sakai H., Sato A., Aihara Y., Ikarashi Y., Midorikawa Y., Kracht M., Nakagama H., Okamoto K. (2014). MKK7 mediates miR-493-dependent suppression of liver metastasis of colon cancer cells. Cancer Sci..

[78] Zhong Y., Naito Y., Cope L., Naranjo-Suarez S., Saunders T., Hong S.M., Goggins M.G., Herman J.M., Wolfgang C.L., Iacobuzio-Donahue C.A. (2014). Functional p38 MAPK identified by biomarker profiling of pancreatic cancer restrains growth through JNK inhibition and correlates with improved survival. Clin. Cancer Res..

[79] Venur V.A., Leone J.P. (2016). Targeted therapies for brain metastases from breast cancer. Int. J. Mol. Sci..

[80] Lee J.Y., Park K., Lim S.H., Kim H.S., Yoo K.H., Jung K.S., Song H.N., Hong M., Do I.G., Ahn T. (2015). Mutational profiling of brain metastasis from breast cancer: Matched pair analysis of targeted sequencing between brain metastasis and primary breast cancer. Oncotarget.

[81] Taglieri L., Nardo T., Vicinanza R., Ross J.M., Scarpa S., Coppotelli G. (2017). Thyroid hormone regulates fibronectin expression through the activation of the hypoxia inducible factor 1. Biochem. Biophys. Res. Commun..

[82] Malik G., Knowles L.M., Dhir R., Xu S., Yang S., Ruoslahti E., Pilch J. (2010). Plasma fibronectin promotes lung metastasis by contributions to fibrin clots and tumor cell invasion. Cancer Res..

[83] Quail D.F., Joyce J.A. (2013). Microenvironmental regulation of tumor progression and metastasis. Nat. Med..

[84] Celia-Terrassa T., Kang Y. (2016). Distinctive properties of metastasis-initiating cells. Genes Dev..

[85] Pulido C., Vendrell I., Ferreira A.R., Casimiro S., Mansinho A., Alho I., Costa L. (2017). Bone metastasis risk factors in breast cancer. Ecancermedicalscience.

[86] Hercbergs A.A., Goyal L.K., Suh J.H., Lee S., Reddy C.A., Cohen B.H., Stevens G.H., Reddy S.K., Peereboom D.M., Elson P.J. (2003). Propylthiouracil-induced chemical hypothyroidism with high-dose tamoxifen prolongs survival in recurrent high grade glioma: A phase I/II study. Anticancer Res..

[87] Hercbergs A., Johnson R.E., Ashur-Fabian O., Garfield D.H., Davis P.J. (2015). Medically induced euthyroid hypothyroxinemia may extend survival in compassionate need cancer patients: An observational study. Oncologist.

[88] Martinez-Iglesias O., Garcia-Silva S., Regadera J., Aranda A. (2009). Hypothyroidism enhances tumor invasiveness and metastasis development. PLoS ONE.

[89] Martinez-Iglesias O., Garcia-Silva S., Tenbaum S.P., Regadera J., Larcher F., Paramio J.M., Vennstrom B., Aranda A. (2009). Thyroid hormone receptor β1 acts as a potent suppressor of tumor invasiveness and metastasis. Cancer Res..

[90] Ruiz-Llorente L., Ardila-Gonzalez S., Fanjul L.F., Martinez-Iglesias O., Aranda A. (2014). microRNAs 424 and 503 are mediators of the anti-proliferative and anti-invasive action of the thyroid hormone receptor beta. Oncotarget.

[91] Huang P.S., Lin Y.H., Chi H.C., Chen P.Y., Huang Y.H., Yeh C.T., Wang C.S., Lin K.H. (2017). Thyroid hormone inhibits growth of hepatoma cells through induction of miR-214. Sci. Rep..

